# Phytosome Technology: A Novel Breakthrough for the Health Challenges

**DOI:** 10.7759/cureus.68180

**Published:** 2024-08-30

**Authors:** Kalaivani P, Kamaraj R

**Affiliations:** 1 Pharmacy, Sri Ramasamy Memorial College of Pharmacy, Chennai, IND; 2 Pharmacy, Sri Ramasamy Memorial Institute Of Science And Technology, Chennai, IND

**Keywords:** phospholipids, bioavailability, target drug delivery, herbosomes, phytosomes

## Abstract

Phytochemicals are compounds found in plants that have various biological activities and health benefits. Although phytochemicals have diverse therapeutic applications, they confront several challenges, such as poor solubility, instability, and low bioavailability. Phytosomes are used to overcome those challenges. The phytosome is a complex of phytochemicals and phospholipids that transports the drug to the target site, thereby increasing phytochemical absorption and bioavailability. The present study focuses on phytosome preparation methods and evaluation parameters, as well as the role of phytosomes in various ailments such as COVID-19, pulmonary fibrosis, asthma, migraine, arthritis, obesity, neuroprotective, antioxidant, anti-inflammatory, cancer, diabetes, metabolic syndrome, hyperlipidemic, and antimicrobial, which demonstrates phytosome complexes are more potent when compared to free extracts. Due to poor absorption and metabolism, phytoconstituents may not be effective in their free form. Phytosomes make phytoconstituents more bioavailable, stable, and effective. It also discusses recent formulations of phytosomes that can act as an effective or alternative regimen for various health conditions.

## Introduction and background

The history of using plants for medicine began when people learned about the beneficial effects of different chemicals derived from herbs. Plants contain various secondary metabolites, including alkaloids, phenolic acids, flavonoids, and terpenoids, which have been shown to have to have therapeutic benefits [1]. Absorption issues limit the bioavailability of biologically active compounds found in plants. To improve bioavailability, herbal products must balance hydrophilic and lipophilic properties. For therapeutic purposes, both traditional and modern medicine systems use plant preparations. Advancements in drug delivery systems, such as targeted drug delivery, have improved the effectiveness of herbal drugs [2]. Phytosomes are lipid-compatible molecular complexes that combine plant extract and phospholipids. Indena, a pharmaceutical and nutraceutical company, created this approach to increase the bioavailability and absorption of plant-derived compounds. According to studies, phytosomes can increase the absorption of chemicals by up to 20 times compared to conventional plant extracts [1]. Phytosomes form in non-polar solvents when the polyphenolic group of phytochemicals forms hydrogen bonds with the phosphate group of phospholipid and when water-soluble polyphenolic compounds interact with the water-containing part of phospholipid [3].

Phytosomes protect herbal extract components from digestive fluids and intestinal microbes. They boost pharmacokinetics and bioavailability, allowing them to pass through lipid-rich biomembranes and into the bloodstream. Phytosomes are important in prolonging the drug's circulation, lowering toxicity, and delaying the clearance of fast-metabolizable medications. Phytosomes exhibit high entrapment capacity because the drug is conjugated with lipids, resulting in stable vesicles that effectively encapsulate the active compounds. Phytosomes offer clinical advantages by improving the therapeutic efficacy and safety profile of herbal extracts. Their enhanced delivery and absorption translate into better clinical outcomes [4]. Phytosomes, also known as herbosomes, are essential in several sectors, such as nutraceuticals, pharmaceuticals, and cosmeceuticals. They offer increased bioavailability and efficacy than standardized extracts, making them useful for a variety of ailments. Popular herbosomes include the Hawthorn herbosome for treating cardiovascular problems; the Ginkgo select herbosome for cognition enhancement and antioxidants; the Casperome boswellia herbosome for inflammatory problems; “Meriva” for joint health; and the Sericoside herbosome to improve wrinkles. Companies that produce and market herbosomes include Indena, Thorne Research, Jamieson Natural Resources, Natural Factors, and Nature Herb. Phytosomes have been employed to increase bioavailability, cure cancer, and promote wound healing [5].

Phytosomes carry the phytochemicals to the target site without the degradation of phytochemicals, increasing the absorption of extracts in the body. A comparison study between free extract and their phytosome complex in various health conditions indicates that phytosomes are effective in drug delivery. Phytosome formulations can serve as an alternate or effective treatment plan for a range of illnesses.

Phospholipids

**Figure 1:**
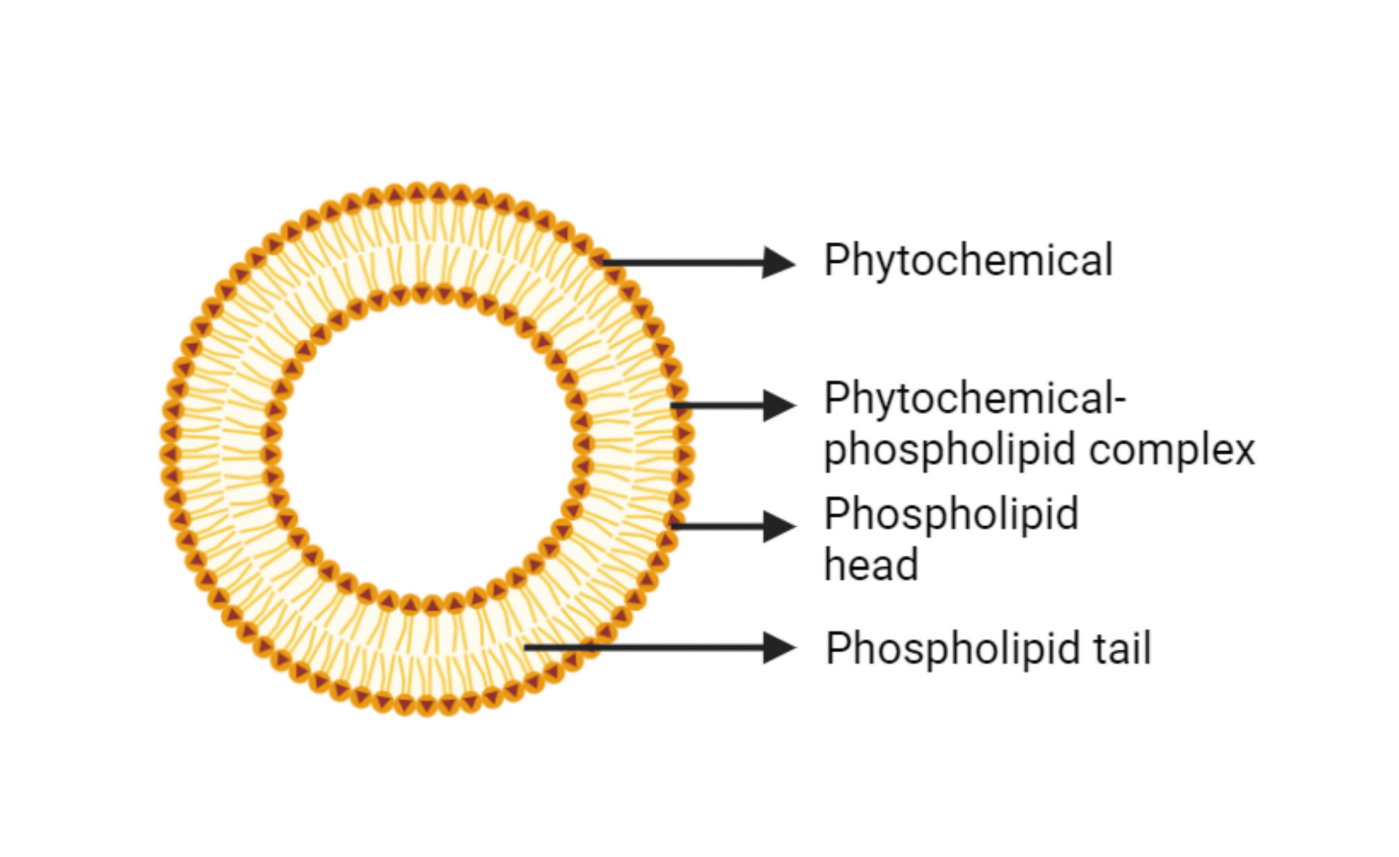
Structure of phytosome Original illustration

Phospholipids, found in cell membranes, can be used as a vehicle for drug administration, making them more flexible. Phospholipids are biocompatible and provide several advantages, including formulation flexibility and the ability to select different novel drug delivery systems based on application. Phospholipids are phosphorus-containing lipids that have polar and non-polar structures [6]. Phospholipids, both natural and synthetic, are commonly used in the pharmaceutical industry. They have a low toxicity profile and can be administered by multiple routes, such as parenteral, oral, and topical. Phospholipids are better excipients than synthetic polymers, which are not appropriate for every route. When selecting natural phospholipids, it is critical to consider the minimum quality of phospholipid content, which differs based on the administration method. Natural phospholipids with at least 45% phosphatidylcholine (PC) can be used for oral and topical administration, but parenteral administration typically requires 70% PC [7]. Human biological cell membranes contain phospholipids such as phosphatidylethanolamine, PC, phosphatidylinositol, phosphatidylserine, and phosphatidic acid. The oxygen atom in the phosphate group of the phytochemical can gain electrons, whereas nitrogen loses electrons, allowing it to be soluble in both the aqueous and lipid phases [6]. The structure of the phytosome is shown in Figure 1.

## Review

Preparation methods of phytosomes

Solvent Evaporation Method

To the round bottom flask, the phytochemical extract and phospholipid were added and heated to 40-50°C with organic solvent under reflux for up to four hours. The resulting mixture was evaporated to 5-10 mL, which was then lyophilized to yield phytosomes. The phytosome was dried, stored in an amber glass container, and refrigerated [8].

Antisolvent Precipitation Method

A phytochemical and phospholipid were taken in a flask containing solvent. The solution was heated below 60°C under reflux for up to two hours, and the mixture was evaporated. n-hexane, known as an antisolvent, was added while stirring in order to get the precipitate. The resultant precipitate was then filtered and kept in a desiccator. The phytosome was then carefully transferred to a light-protective container and refrigerated [9].

Rotary Evaporation Method

The phytochemicals and phospholipids were mixed with tetrahydrofuran. The resulting mixture was then agitated for three hours at temperatures less than 40°C, and n-hexane was added to the mixture after the formation of a thin film. It was then mixed with the help of a magnetic stirrer. The resultant precipitate was transferred to an amber glass container and refrigerated [9].

Evaluation of phytosome

Visualization

The samples can be visualized using transmission electron microscopy (TEM) and scanning electron microscopy (SEM). The samples are sputter-coated with palladium or gold for 120 seconds at 14 mA in an argon atmosphere for the ultra-thin application of metals on the sample, and the sputter-coated samples are mounted on the microscope to observe the particle size and surface morphologies of the sample. Smaller particle sizes have larger surface areas, which increases the absorption across biological membranes [8].

Zeta Potential

Zeta potential is the measure of the surface charge of the phytosome. It is a crucial parameter that impacts the stability and performance of the final product. A zeta sizer with an argon laser is used. Before mounting the sample onto the zeta sizer, dilute it with the solvent to analyze the zeta potential of the phytosome complex. A higher zeta potential value indicates that phytosomes remain stable over time. It is expressed in millivolts (mV) [2].

Entrapment Efficiency

Entrapment efficiency is defined as the percentage of active compounds entrapped in the vesicles. The amount of entrapment of phytosomes can be estimated by diluting them with methanol and subjecting them to centrifugation at 10,000 rpm for 30 minutes at -40°C on a cooling centrifuge. The supernatant is collected, and the quantity of entrapped extract is determined using a UV spectrophotometer. Higher entrapment efficiency can enhance the stability and bioavailability and control the release of the phytochemicals. It can be calculated using the following formula [10].

Entrapment efficiency (%) = total amount of drug - amount of free drug / total amount of drug × 100

Differential Scanning Electroscopy

To determine the compatibility and thermal stability of the sample, the extract, phospholipid, their combination, and the phytosome complex are heated at 50-250°C per minute, from 0 to 400°C, using an aluminium cell in a nitrogen atmosphere. A differential scanning calorimetry (DSC) thermogram can determine the temperature at which the phytosome decomposes or undergoes phase changes. It also measures the phase transitions of the samples [11].

Fourier-Transform Infrared Spectroscopy (FT-IR)

To determine the molecular interactions between the extract and phospholipids, the potassium bromide method (KBr) is used to obtain the infrared spectra of free extract, phospholipid, a combination of extract and phospholipid, and their phytosome complex. To make KBr pellets, a sample was added to KBr at a 1:100 ratio, and the sample pellets were analyzed between 4000 and 400 cm-1 [12].

Stability Study

As per the International Council for Harmonisation of Technical Requirements for Pharmaceuticals for Human Use (ICH) guidelines, the stability study of the phytosome was conducted for a duration of three months at a temperature and relative humidity of 40 ± 2°C / 75 ± 5% and at 30 ± 2°C / 60 ± 5% [13].

Role of phytosome in various diseases

COVID-19

The early phase of COVID-19 starts with an immune response, followed by a second phase where an activation of macrophages and a cytokine storm occur. Quercetin can inhibit proteins involved in the COVID-19 infection cycle. Rondanelli et al. conducted a pilot study to study the effectiveness of the Quercetin Phytosome® in preventing COVID-19. The researchers selected 120 subjects, divided them into two groups, and classified 60 subjects as the supplementation group and another 60 as the control group. The supplementation group received 250 mg of Quercetin Phytosome® twice daily for three months. Every three weeks, the subjects had a rapid COVID-19 diagnosis. Out of the study participants, there was one case of COVID-19 in the group receiving supplementation, whereas the control had four cases. The study found that subjects under supplementation improved their symptoms at seven and 15 days, as well as a 99.8% COVID-free survival rate at five months in the quercetin group and 96.5% in the placebo group. The subjects who received the Quercetin Phytosome® supplement experienced a 14% greater protective effect against COVID-19 compared to those who received the placebo. The results demonstrated the effectiveness of Quercetin Phytosome® against COVID-19, but more research is necessary to incorporate it as a regular preventive measure [14].

Pulmonary Fibrosis

Idiopathic pulmonary fibrosis is a deadly and incurable lung disease. Researchers discovered that hydroxysafflor yellow A (HSYA), a polar compound in safflower, prevents TGF-1 from activating lung fibroblasts. Tingting et al. researched the HSYA phytosome (HYAP) through intervaginal space injection (ISI) to examine its impact on lung fibrosis. The researchers developed HYAP using the thin film method to enhance the solubility and bioavailability of HSYA. They tested it in several ways, including TEM, dynamic light scattering (DLS), entrapment efficiency, UV analysis, and FT-IR. They then injected bleomycin (BLM) (5 mg/kg) into mice to induce pulmonary fibrosis. After seven days, researchers separated the animals into three categories: saline, standard, and test. The standard group received pirfenidone (200 mg/kg) for up to 14 days, while the test group received HYAP (120 mg/kg) in 0.1 mL of saline solution through ISI for the same period. Fibrous tissue growth, collagen deposition, and inflammatory response have diminished in the standard and test groups (HYAP) compared to the BLM group. MAPK-p38 and the TGF-β/Smad pathway play a critical role in the progression of pulmonary fibrosis. The inhibited pathways in the standard and HYAP groups indicate that HYAP can be an alternative regimen for pulmonary fibrosis management [15].

Asthma

Ferrara et al. investigated the safety and efficacy of Casperome® in reducing the need for inhalational therapy. Boswellia serrata produces Casperome®, a phytosome that can treat asthma. The study enrolled 32 patients. The researchers assigned 18 subjects to the Casperome® group and classified 14 subjects as the control group. During the study, the patients took inhalational therapy two times a day to evaluate the reduction in frequency between the control group and the treatment group. After four weeks, the Casperome® group experienced a 43% reduction in inhaler use. Nineteen patients have reported adverse events out of 32 patients, eight from the Casperome® group and 11 from the control group. The study found that moderate adverse events occurred, with no serious problems reported [16].

Migraine

Curcumin can be effective in treating migraine attacks, but its efficacy is limited due to poor absorption. Researchers can develop phytosomal curcumin to enhance its absorption. Shojaei et al. carried out a clinical trial with 60 subjects suffering from migraine. The test group received phytosomal curcumin (250 mg) for eight weeks, while the control group received maltodextrin (250 mg) for the same period. The researchers took a blood sample both before and after the 12-hour treatment period, using enzyme-linked immunosorbent assay (ELISA) to measure total antioxidant capacity, malondialdehyde, high-sensitivity C-reactive protein, and nitric oxide. Because phytosomal curcumin is highly bioavailable and can cross the blood-brain barrier, it lowers oxidative stress and neuroinflammation, which in turn lowers migraine in patients [17].

Arthritis

Ramezani et al. assessed the effect of phytosomal curcumin on collagen-induced arthritis in rats. The researchers divided 40 male Wistar rats into five groups. In rats, phytosomal curcumin reduced paw swelling, and histopathological analysis showed less joint damage and inflammatory infiltration. It also decreased the expression of pro-inflammatory cytokines like tumor necrosis factor-alpha (TNF-α), IL-17A, and matrix metalloproteinase-8 (MMP-8) mRNA while increasing the expression of anti-inflammatory cytokines like IL-10 and TGF-β. The Th17 cell response decreased, while the Treg cell response increased, compared to the control group. The study suggests that phytosomal curcumin can be beneficial due to its increased bioavailability and immunomodulatory properties for the treatment of arthritis, but additional studies are required to confirm its potency and safety [18].

Obesity

Ortega-Pérez et al. prepared Callistemon citrinus-loaded phytosomes for obesity management. They prepared the phytosome using the thin-layer sonication method and evaluated it based on particle size and entrapment efficiency. The phytosome can remain stable at 20ºC for three and a half months. An in vivo study on obesity demonstrated that the Callistemon citrinus phytosome can decrease body weight by 40% in rats with high fat. They identified ellagic acid in Callistemon citrinus for the first time [19].

Neuroprotective

Mane et al. developed Emblica officinalis phytosomes (EOP) to improve the bioavailability, solubility, and cognitive functions of Emblica officinalis extract (EOE). Using the solvent-evaporation method, they developed phytosomes. Then, to make the process better, they used a Box-Behnken design and a UV spectrophotometer set to 275 nm to check the solubility and in vitro drug release studies of EOP and EOE. They also conducted a six-month stability study on EOP under accelerated conditions. In the elevated plus maze and Morris water maze tests, EOP enhanced memory more than EOE, with higher dopamine, serotonin, and acetylcholine concentrations. The result showed that EOP had better neuroprotective activity due to its higher bioavailability and absorption than plain EOE [20].

Antioxidant and Anti-inflammatory

By using the thin layer hydration method, Deleanu et al. developed Phytoginrosa (PGR), which combines ginger and rosehip to increase bioavailability, anti-inflammatory, and antioxidant benefits. PGR, at a ratio of 0.5:0.5:1, is the most effective formulation, with an increase in antioxidant enzymes and inhibition of pro-inflammatory molecules. The optimized phytosome increased the synergistic activity of ginger and rosehips, indicating that they were more effective and safer for long-term use than individual constituents [21].

By using the solvent evaporation method, Solanki et al. developed phytosomes loaded with Tabernaemontana divaricata to make them more antioxidant. They conducted a DPPH (2,2-diphenyl-1-picrylhydrazyl) assay to determine the antioxidant activity of phytosomes. The result demonstrated that the obtained IC50 values were comparable between the free extract and the phytosome. Researchers confirmed that phytosomes loaded with extract can enhance the extract's bioavailability, half-life, and antioxidant content [22].

Cancer

When compared to a free extract of phytochemicals, the phytosomes are effective. The role of phytosomes in cancer is tabulated in Table 1 [23-26].

**Table 1:** Phytosomes role in types of cancer CUR - curcumin; G1 phase - gap 1 phase; G2 phase - gap 2 mitosis phase; IC50 - half maximal inhibitory concentration; MOPP - *Moringa oleifera* leaf polyphenol; NF-kB - nuclear factor kappa-light-chain-enhancer of activated B cells; PL-SV - Phospholipon® 90H - scorpion venom; S phase - synthesis phase; THQ - thymoquinone; TNF-α - tumor necrosis factor-alpha

Types of cancer	Components	Particle size and zeta potential	IC50	Cell cycle arrest	Gene expression	Inflammatory markers
Breast cancer (MCF-7 cell line)	Quercetin, scorpion venom peptides, Phospholipon® 90H	116.9 nm and 31.5 mV	Lower than plain and quercetin	S phase	Increased caspase-9, Bax	Reduced TNF-α, NF-kB
Lung adenocarcinoma (A549 cell line)	Thymoquinone, scorpion venom peptides, Phospholipon^®^ 90H	209.9 nm and 21.1 mV	Lower than plain and THQ	S phase	Increased caspase-3, Bax, and p53	Reduced TNF-α, NF-kB
Breast cancer (4T1 cell line)	*Moringa oleifera*, soy phosphatidylcholine	296 ± 0.29 nm and 296 ± 0.29 nm	Lower than MOPP extract	-	-	-
Prostate cancer (PC3 cell line)	Curcumin, scorpion venom peptides, Phospholipon^®^ 90H	137.5 ± 7.9 to 298.4 ± 11.9 nm and 2.9 ± 0.1 to 26.9 ± 1.2 mV	Lower than CUR and PL-SV	Pre-G1 and G2-M phases	Increased Bcl-2 and reduced Bax, p53, caspase-3	Reduced TNF-α, NF-kB

Diabetes

Singh et al. focused on the preparation and characterization of phytosomes that combine Swertia chirata and Zizyphus mauritiana. They prepared the phytosomes using solvent evaporation, antisolvent, and rotary evaporation methods. The study demonstrated the effectiveness of an antisolvent precipitation method due to its lower particle size and greater entrapment efficiency. Researchers who looked at phytosomes in vitro found that the Korsmeyer-Peppas model fits them best. Higher regression values were found for drug release by diffusion in the body. The extract loaded with phytosomes has a higher release rate compared to the plain extract. In vivo studies demonstrated that the phytosomes were less effective than extracts in decreasing blood glucose levels. Thus, the phytosome did not exhibit better antidiabetic activity in the streptozotocin-induced model [9].

Hepatoprotective

Sharma et al. investigated the effectiveness of plumbagin phytosomes for hepatoprotective activity. They used the antisolvent precipitation method and evaluated it based on particle size, entrapment efficiency, and percentage yield. They carried out pre-formulation studies to find out the physiochemical properties of plumbagin and an in vitro dissolution study to determine how the drug would be released in the body [27].

Metabolic Syndrome

Palachai et al. developed the phytosome with a combined extract of mulberry and ginger (PMG) for the management of metabolic syndrome (MetS). In rats, PMG enhanced metabolic parameters by lowering body weight, plasma glucose levels, and inflammatory cytokines while simultaneously boosting peroxisome proliferator-activated receptor gamma (PPAR-γ), lipid profiles, and insulin resistance. These results showed that PMG is an effective approach for MetS treatment [28].

Hyperlipidemic

Dudekula et al. developed guggulsterone phytosomes (GPs) using the solvent evaporation method. This includes evaluating GPs for particle size, zeta potential, and entrapment efficiency. An MTT (3-(4,5-dimethylthiazol-2-yl)-2,5 diphenyl tetrazolium bromide) assay showed that GPs had better compatibility and reduced cell viability. Rats treated with GPs had lower body weight, total cholesterol, triglycerides, LDL, and atherogenic index, which indicates a lower risk of atherosclerosis compared to the control group. Thus, the study confirmed that the prepared GPs had increased the hypolipidemic activity of guggulsterone [29].

Antimicrobial

Jagtap et al. developed a hydroalcoholic extract of Adiantum capillus-veneris (ACV) phytosomes using the antisolvent precipitation method for antimicrobial activity. The researchers evaluated the ACV extract's phytosome using encapsulation efficiency, particle size, zeta potential, dissolution, and compatibility studies. The phytosomal formulation was more effective because the particles were smaller, and the phytosomes were able to penetrate deeper than the free hydroalcoholic extract [30].

Formulation of phytosomes

The various dosage forms of phytosomes are mentioned in Table 2 [11,31-54].

**Table 2:** Formulations of phytosomes

Dosage form	Material	Method	Particle size	Zeta potential	Entrapment efficiency	Indication
Tablet	*Cucumis sativus* linn, soya lecithin	Antisolvent precipitation method	332 ± 0.4 nm	-16.85 mV	89.49%	-
	*Mangifera indica*, soya lecithin		452.88 ± 0.5 nm	-19.40 mV	91.5%	-
Capsule	*Physalis minima* linn, soya lecithin	Solvent evaporation method	254 nm	-7.57 mV	51%	-
	*Mucuna prureins*, soya lecithin		216.3 ± 14 nm	-37.45± 0.2 mV	99.76 ± 1.24%	-
	Curcumin, soy phosphatidylcholine		150-200 nm	-35.4 ± 2.45 mV	93.35%	-
Cream	*Phyllanthus emblica*, soy phosphatidylcholine		210.5 nm	-36.7 mV	94.03 ± 0.10%	Photo protectant
	*Polygonum hydropiper*, *Cuscuta reflexa*, *Flemingia strobilifera*, *Azadirachta indica*, *Cymbopogon nardus*, phosphatidylcholine	Rotary evaporation method	547 ± 1.74 nm	-	94.03 ± 0.10%	Black fly bites
	*Mahonia aquifolium*, white willow bark, *Aloe vera*, soya lecithin		-	-	-	Psoriasis
	*Curcuma longa* and *Arnica montana*, L-α-phosphatidylcholine	Thin film hydration method	214.8 to 280.1 nm	+26 mV	>80%	Cold injuries
	Orange peel extract and licorice extract, phosphatidylcholine	Solvent evaporation method	-	-	93.22 ± 0.26%	Skin aging
Gel	*Azadirachta indica*, phosphatidylcholine, cholesterol	-	676 ± 0.007 nm	-	98.20 ± 0.059%	-
	*Quercus infectoria*, soya lecithin, cholesterol	Solvent Evaporation method	-	-	-	Antimicrobial
	*Camellia sinensis*, Lipoid P30	Thin layer hydration method	-	-	-	Anti-aging
	*Aegle marmelos*, soya lecithin	Antisolvent precipitation method	-	-	89.25%	-
	Resveratrol, phosphatidylcholine, cholesterol	Rotary evaporation method	521 to 676 nm	-24.6 to 28.5 mV	86 to 98.2%	-
Hydrogel	Quercetin dihydrate, soybean hydrogenated phosphatidylcholine	Antisolvent precipitation method	400 to 450 nm	30 mV	>80%	-
Lotion	*Nothopanax scutellarium*, Phospholipon® 90 G	Thin film hydration method	289 ± 1.41nm	-9.10 ± 1.5 mV	99.76 ± 0.24%	Hair growth
	*Berberis aquifolium*, soya lecithin	Rotary evaporation method	-	-	-	Eczema
Face serum	*Carica papaya*, *Zingiber officinale*, soya lecithin, phosphatidyicholine	Antisolvent precipitation method	198.09 ± 0.04 nm	-27.8 mV	-	Antioxidant, antimicrobial, moisturizing
	*Vitis vinifera*, phosphatidylcholine	Thin layer hydration method	398.23 nm	-25.2 mV	75.01 ± 0.25%	Anti-aging
	Cocoa pod husk, phosphatidyicholine		627 nm	-	90.05%	Antioxidant, tyrosinase inhibitor
Phytosome microsphere	*Camellia sinensis*, Lipoid P 30, maltodextrin, gum arabica	Thin layer hydration method	-	-48.2 ± 1.78 mV	50.61 ± 0.93%	Antioxidant
	Allicin-rich extract, soya lecithin, Eudragit® L30D-55	Spray dry method	251.6 nm	34.11 mV	62.62 %	-
Liquid oral	Lycopene, phosphatidylcholine	Solvent evaporation method	450.5 to 594.5 nm	-21.2 to -23.2 mV	-	Antioxidant
Nasal vaccine	Diammonium glycyrrhizinate, phosphatidylcholine	Solvent evaporation method	20-30 nm	-30 to -40 mV	-	Immune-enhancer

## Conclusions

Many phytochemicals have the potential to fight diseases, but their physiochemical properties make them less effective. Phytosomes are the best way to deliver drugs because they make phytochemicals more soluble and improve pharmacokinetic parameters. They also keep bioactives from being broken down by enzymes and lower the dose and frequency of drugs. Phytosomes help phytochemicals overcome hurdles. It can be formulated into different dosage forms, such as tablets, capsules, suspensions, creams, and gels, depending on the disease and phytochemicals. As a result, the phytosomes have significant potential for developing effective formulations that lead to improved patient outcomes and an alternative treatment regimen. In the future, phytosomal formulations for the endocrine system, including thyroid and uterine functions, could be developed, paving the way for effective treatment for managing endocrine disorders.
